# Population pharmacokinetics of rifabutin among HIV/TB co-infected children on lopinavir/ritonavir-based antiretroviral therapy

**DOI:** 10.1128/aac.00354-24

**Published:** 2024-07-22

**Authors:** Manna Semere Gebreyesus, Roeland E. Wasmann, Helen McIlleron, Regina Oladokun, Prosper Okonkwo, Lubbe Wiesner, Paolo Denti, Holly E. Rawizza

**Affiliations:** 1 Division of Clinical Pharmacology, Department of Medicine, University of Cape Town, Cape Town, South Africa; 2 Department of Pediatrics, Faculty of Clinical Sciences, College of Medicine, University of Ibadan, Ibadan, Nigeria; 3 APIN Public Health Initiatives (APIN), Abuja, Nigeria; 4 Brigham and Women’s Hospital, Boston, Massachusetts, USA; 5 Harvard T.H. Chan School of Public Health, Boston, Massachusetts, USA; University Children's Hospital Münster, Münster, Germany

**Keywords:** pediatric, infectious diseases, drug interactions, rifabutin, antiretroviral therapy

## Abstract

In adults requiring protease inhibitor (PI)-based antiretroviral therapy (ART), replacing rifampicin with rifabutin is a preferred option, but there is lack of evidence to guide rifabutin dosing in children, especially with PIs. We aimed to characterize the population pharmacokinetics of rifabutin and 25-O-desacetyl rifabutin (des-rifabutin) in children and optimize its dose. We included children from three age cohorts: (i) <1-year-old cohort and (ii) 1- to 3-year-old cohort, who were ART naïve and received 15- to 20-mg/kg/day rifabutin for 2 weeks followed by lopinavir/ritonavir (LPV/r)-based ART with 5.0- or 2.5 mg/kg/day rifabutin, respectively, while the (iii) >3-year-old cohort was ART-experienced and received 2.5-mg/kg/day rifabutin with LPV/r-based ART. Non-linear mixed-effects modeling was used to interpret the data. Monte Carlo simulations were performed to evaluate the study doses and optimize dosing using harmonized weight bands. Twenty-eight children were included, with a median age of 10 (range 0.67–15.0) years, a median weight of 11 (range 4.5–45) kg, and a median weight-for-age *z* score of −3.33 (range −5.15 to −1.32). A two-compartment disposition model, scaled allometrically by weight, was developed for rifabutin and des-rifabutin. LPV/r increased rifabutin bioavailability by 158% (95% confidence interval: 93.2%–246.0%) and reduced des-rifabutin clearance by 76.6% (74.4%–78.3%). Severely underweight children showed 26% (17.9%–33.7%) lower bioavailability. Compared to adult exposures, simulations resulted in higher median steady-state rifabutin and des-rifabutin exposures in 6–20 kg during tuberculosis-only treatment with 20 mg/kg/day. During LPV/r co-treatment, the 2.5-mg/kg/day dose achieved similar exposures to adults, while the 5-mg/kg/day dose resulted in higher exposures in children >7 kg. All study doses maintained a median *C*_max_ of <900 µg/L. The suggested weight-band dosing matches adult exposures consistently across weights and simplifies dosing.

## INTRODUCTION

In 2021, it was estimated that 185,000 tuberculosis (TB)-related deaths occurred among people with human immunodeficiency virus (HIV), of which 11% were children (1). The ratio of TB-related mortality to incidence was 0.35 in children under the age of 15 with HIV, higher than 0.19 in children without HIV (2). According to Clinton Health Access Initiative reports, in 2022, 24% of children with HIV were on lopinavir/ritonavir (LPV/r)-based antiretroviral (ART) regimen (3). World Health Organization (WHO) guidelines indicate that LPV/r-based ART is recommended as an alternative first-line regimen for children >3 kg aged from 4 weeks to 10 years and a preferred second-line regimen for all children and adolescents who have failed dolutegravir-based first-line ART (4). Among adults undergoing LPV/r-based ART, use of rifabutin is recommended instead of rifampicin, since rifampicin decreases the concentration of lopinavir by more than 75%, while rifabutin has no significant effect on LPV/r exposure (5–10). On the other hand, CYP3A4 contributes to the elimination of rifabutin and 25-O-desacetyl rifabutin (des-rifabutin), its metabolite from arylacetamide deacetylase (AADAC) (11). Thus, when co-administered with inhibitors of CYP3A4 such as LPV/r, exposures of both rifabutin and des-rifabutin are increased (12). This may result in adverse effects such as neutropenia, thrombocytopenia, arthralgias, skin discoloration, and anterior uveitis (13–15), and necessitates dose adjustment of rifabutin during co-treatment as well as monitoring of adverse effects.

During LPV/r therapy in adults, it is recommended that rifabutin dose be reduced to 50%–75% of the standard dose (16). Accordingly, the adult rifabutin dose is decreased from 300 to 150 mg/day when co-administered with LPV/r, as studies have demonstrated comparable exposure levels without impairing exposures of LPV/r (7, 10). In children, rifabutin is recommended at 5 mg/kg/day for prophylaxis of *Mycobacterium avium* complex (MAC) and at 10–20 mg/kg/day for MAC and TB treatment (17). However, there is a lack of data in children regarding the optimal dose during LPV/r-based antiretroviral therapy (ART). A study in children administered rifabutin alongside LPV/r by Moultrie et al. involved six children and was halted due to the occurrence of grade 4 neutropenia in two of the children (15).

In this work, a population pharmacokinetic analysis was performed using data from three age cohorts of children with HIV/TB co-infection who underwent rifabutin-based TB treatment along with LPV/r-based antiretroviral therapy. The objectives were to characterize the pharmacokinetics of rifabutin and des-rifabutin, to perform simulations to assess adequacy of dosing in the studies, and to devise a weight band-based dosing regimen to align pediatric exposure levels with those achieved in adults undergoing standard TB treatment.

## MATERIALS AND METHODS

### Study participants

Data for this study were obtained from three prospective studies carried out in Nigeria in children who were part of the AIDS Pevention Initiative in Nigeria through the US President’s Emergency Plan for AIDS Relief (APIN PEPFAR) pediatric ART program and required protease inhibitor-based ART. The studies included three distinct age groups: <1 year old, 1–3 years old, and 3–15 years old.

### Drug administration and sampling

A <1-year cohort initially underwent TB treatment that included rifabutin at a dose of 20 mg/kg/day, following the US guidelines for treating opportunistic infections in children with and exposed to HIV (17). After 2 weeks, they began LPV/r-based ART, at which point the rifabutin dose was adjusted to 5 mg/kg/day. Similarly, a 1- to 3-year-old cohort received a 2-week TB regimen with rifabutin at a dose of 15–20 mg/kg/day, followed by initiation of LPV/r-based ART, with the rifabutin dose adjusted to 2.5 mg/kg/day. A 3- to 15-year-old cohort was already experienced with ART and was failing on the first-line regimen, necessitating a switch to LPV/r-based ART. On the same day as the switch, they were included in the study and began TB treatment with a rifabutin dose of 2.5 mg/kg/day (18).

The rifabutin dose during LPV/r-based ART for all age cohorts in the study was decided based on extrapolation from adults with adjustments based on allometry for children and knowledge gained from the study by Moultrie et al., where adequate concentrations were reported after a 5-mg/kg/day thrice weekly dose during LPV/r co-treatment (15, 18–20). Rifabutin was administered as an oral suspension prepared from Mycobutin capsules by Lupin Pharmaceuticals, as previously detailed (21).

Blood samples were collected intensively at 0, 2, 4, 8, 12, and 24 h post-dose during weeks 2 and 4 for all age groups. Additionally, in the cohort of children under 1 year old, samples were taken at week 6, and in the 3- to 15-year-old cohort, samples were collected at week 8. Sparse samples were obtained at weeks 6 and 12 (at 0 and either 3–5 or 24–26 h post-dose) in the 1- to 3-year-old cohort. The intensive samples at week 2 were obtained following rifabutin-containing TB-only treatment in the <1-year and 1- to 3-year groups, while the week 2 intensive samples for the 3- to 15-year group, who had experience with ART, and samples from all other visits were taken after rifabutin with LPV/r co-treatment. Specifically, data on rifabutin only were available for the two younger cohorts, while data on rifabutin with LPV/r were available across all age groups. A schematic of the study design is included in Fig. S1.

Data from the Moultrie et al. study were used for external model validation (15). This study was conducted in South African children with HIV ≤5 years old, receiving LPV/r-based ART with rifabutin dosed at 5 mg/kg/day thrice weekly. Blood samples were collected after the sixth dose at 0, 2, 4, 9, 24, and 48 h. After validation, these data were incorporated into the analysis, and the parameters were re-estimated.

### Drug quantification

All samples were centrifuged at 2,600 rpm for 10 min to extract plasma within 1 h after collection and were then stored at −80°C until analysis. Rifabutin and des-rifabutin concentrations were concurrently quantified at the University of Cape Town with a validated liquid chromatography-tandem mass spectrometry assay with a calibration range of 3.91–1,000.0 µg/L for rifabutin and 0.780–200 µg/L for des-rifabutin (15).

### Population pharmacokinetic analysis

A joint population pharmacokinetic model for both rifabutin and des-rifabutin was developed using non-linear mixed-effects modeling in NONMEM (v.7.5.0). First-order conditional estimation with eta-epsilon interaction was used for all model runs. Model management was done with Pirana (v.2.9.8), and post-processing of results and visualization of output were carried out with Perl-speaks-NONMEM (v.5.2.6) and R (v.4.3.0) (22). The modeling process was sequential, starting with the development of the parent (rifabutin) model, followed by development of the metabolite (des-rifabutin) model while maintaining fixed population parameter estimates for the parent, then proceeding with the joint parent-metabolite model. Various disposition models were tested for both the parent and the metabolite, including one-, two-, and three-compartment models, incorporating first-order absorption and elimination, along with the effect of LPV/r co-treatment. To describe absorption delay, lag time and transit compartments were evaluated. Allometric scaling using total body weight was applied to all disposition parameters with allometric exponents fixed to 0.75 and 1.0 for clearances and volumes of distribution, respectively (19).

Molar conversion of the rifabutin dose and the concentrations of rifabutin and des-rifabutin was utilized to adjust for the difference in molecular weight between rifabutin (847.02 g/mol) and des-rifabutin (805 g/mol). Since it is generally not possible to estimate both the fraction metabolized (*F*_M_) and the metabolite disposition parameters concurrently in joint parent metabolite models, three approaches were tested to address the challenge of model identifiability (23):

1. Assuming full conversion of the parent to the metabolite (*F*_M_ fixed to 1).
2. Assuming volumes of distribution for the parent and metabolite are equivalent and estimating the volumes as well as *F*_M_.
3. Estimating metabolite volumes and clearance conversion to the metabolite with a covariate on the clearance of the parent via an alternative pathway.

In certain pharmacokinetic profiles, it was observed that both the rifabutin and des-rifabutin concentrations just prior to the observed dose were dramatically lower than typically expected at steady state for a drug with a long terminal half-life like rifabutin, when the pre-dose and 24-h concentrations should remain fairly similar. To address this issue, whenever a pre-dose concentrations fell below one-third of the concentrations 24 h after the observed dose, the prior dosing history was disregarded and the baseline method B2 was used (24). This approach initializes all disposition compartments using the observed concentration values and the residual unexplained variability (more details the supplemental material).

Between-subject and between-occasion random effects were estimated, assuming they follow a log-normal distribution. Combined error model (i.e., additive and proportional error) was used to describe the unexplained residual variability. Additionally, considering that both rifabutin and its metabolite were quantified simultaneously and from the same sample, a correlation coefficient between their residual unexplained variability terms was incorporated using the L2 method (25). Samples below the lower limit of quantification (LLOQ) [below the limit of quantification (BLQ)] were received as censored and handled using a variant of the M6 method by Beal (26); i.e., 50% of the LLOQ was imputed for the last BLQ during absorption and the first BLQ during elimination, and their additive error was increased by LLOQ/2. All other BLQs in a series were excluded from the model fit but retained for model diagnostics. Model development and selection were based on inspection of diagnostic plots and statistical significance. This latter aspect was evaluated with changes in the objective function value (OFV), whereby for two nested models, an additional degree of freedom (i.e., one extra estimated parameter) is deemed statistically significant at *P* < 0.05 if the improvement in OFV is at least 3.84 points. For non-nested models, the Akaike information criterion (AIC) was used to assess goodness of fit.

Screening for covariates was performed by inspecting plots of individual empirical Bayes estimates vs covariates and was scrutinized based on physiological plausibility. Covariates tested in the model included LPV/r co-treatment, maturation (27), weight-for-age *z* score (ZWFA, calculated using WHO growth charts for ≤10 years old and Centers for Disease Control and Prevention growth charts for >10 years old), CD4 count, age group, and creatinine clearance (calculated by the modified Schwartz equation) (28). These were tested individually in the model and selected using a stepwise approach with *P* < 0.05 on forward addition and *P* < 0.01 on backward elimination. Area under the concentration-time curve (AUC)_0–24 h_ and *C*_max_ were derived from the final model, using the individual post hoc pharmacokinetic parameters, during the intensive sampling periods with and without LPV/r co-treatment.

Model performance was assessed throughout model development using a prediction-corrected visual predictive check (pcVPC) since there were several doses and a wide weight range in the overall data (29). Parameter precision of the final model was obtained using sampling importance re-sampling procedure (30).

### Simulations

Monte Carlo simulations were performed with the final model, including random effects parameters, to evaluate the doses used in the study and suggest weight-banded rifabutin pediatric dosing during LPV/r co-treatment using harmonized weight bands (31). For these simulations, we used a representative *in silico* population of 22,500 African children (32) with uniformly distributed weight. A sample size of 500 children per unit kilogram was used for the simulations. Steady-state AUC_0–24 h_ and *C*_max_ were obtained for two dosing scenarios:

1. Milligrams per kilogram dosing (replicating the doses used in the studies).
  - Without LPV/r co-treatment: for a scenario of TB-only treatment, rifabutin dose of 20 mg/kg/day was simulated. In the study, the 3- to 15-year-old cohort did not receive TB-only treatment, but this scenario was simulated for all the virtual pediatric populations with the rifabutin dose capped to 300 mg/day, which is the adult dose.
  - With LPV/r co-treatment: during TB-HIV co-treatment, rifabutin dose of 5 mg/kg/day was simulated for the <1-year-old cohort and 2.5 mg/kg/day was simulated for the ≥1-year-old cohort, similar to the study.
2. Harmonized weight band-based dosing.
  - Rifabutin doses optimized for scenarios without and with LPV/r co-treatment were simulated for the following harmonized weight bands (31): ≥6 to 10 kg, ≥10 to 15 kg, ≥15 to 20 kg, ≥20 to 25 kg, ≥25 to 30 kg, ≥30 to 35 kg, and ≥35 kg.

### Targets for simulations

Rifabutin lacks clear reports defining its therapeutic range, but a relationship between the AUC and the efficacy of antituberculosis drugs has been suggested (33, 34). Other sources propose that *C*_max_ exceeding 900 µg/L could elevate the risk of adverse reactions (35–37). In line with this, we compared the medians of our simulated exposures (AUC_0–24 h_) with a range of reported median AUC_0–24 h_ values in adults and evaluated attainment of *C*_max_ of <900 µg/L. For standard TB treatment [300 mg once daily (OD) rifabutin dose in adults], our comparator adult median AUC_0–24 h_ values varied from 5,640 µg·h/L (Lan et al.) to 2,790 µg·h/L (Tanuma et al.) for rifabutin and from 700 µg·h/L (Lan et al.) to 273 µg·h/L (Naiker et al.) for des-rifabutin (9, 10, 38). During co-treatment with LPV/r (150-mg OD rifabutin dose in adults), the median AUC_0–24 h_ for rifabutin ranged from 7,290 µg·h/L (Lan et al.) to 4,770 µg·h/L (Naiker et al.) and from 4,130 µg·h/L (Lan et al.) to 4,120 µg·h/L (Naiker et al.) for des-rifabutin (9, 10). Furthermore, for dose optimization with weight band-based dosing, the aim was to achieve a median steady-state AUC_0–24 h_ of at least 4,500 µg·h/L, an exposure limit reported to be associated with acquired rifamycin resistance (37, 39, 40).

## RESULTS

### Participant characteristics

The study included 28 children living with HIV and TB co-infection who were enrolled in the APIN PEPFAR program in Nigeria. Baseline characteristics of the participants are presented in Table 1. Three children were less than 1 year old; 10 were aged 1–3 years; and 15 were aged 3–15 years. The overall median age was 10 (range 0.67–15.0) years, and the median weight was 11 (range 4.5–45.0) kg. ~60% of the children were severly underweight (ZWFA < –3), with an overall ZWFA of −3.33 (range −5.15 to –1.32). In the oldest cohort (3–15 years old), the children had been on ART for a median of 3.8 (range 1.5–8.9) years, with 4 out of 15 classified as WHO HIV stage 4, while the remaining were at stage 3. Overall, neutropenia was observed in 12 children on 26 occasions (10 occasions: grade 1, 6 occasions: grade 2, 8 occasions: grade 3, and 2 occasions: grade 4). A total of 462 samples were available for quantification of rifabutin and des-rifabutin, and only 16 samples (1.7%) were BLQ. Fifteen profiles had pre-dose concentrations lower than one-third of 24-h concentrations across different visits (Fig. S3) and were handled by the method of initialization, detailed in the supplemental material.

**TABLE 1:** Patient demographic and study characteristics, presented as median (range) where applicable^*c*^

Study cohort	<1 year old *n* = 3	1–3 years old *n* = 10	3–15 years old (18) *n* = 15	Validation data (15) *n* = 6
ART duration (years)	ART-naïve	ART-naïve	3.8 (1.5–8.9)	1 (0.5–2.3)
Backbone ART	ABC + 3TC	ABC + 3TC	ABC + 3TC	ABC/d4T + 3TC
Age (months)	11 (8–11)	18 (14–28)	157 (123–185)	25 (10–41)
Weight (kg)	4.75 (4.50–6.50)	7.33 (5.60–11.0)	27.0 (20.0–45.0)	10.6 (8.50–12.2)
Height (cm)	75 (71–78)	73 (70–84)	138 (121–164)	79 (74–90)
ZWFA^*a*^	−4.95 (−5.15 to −3.20)	−2.64 (−5.06 to −1.39)	−3.25 (−4.38 to −1.32)	−1.06 (−1.85 to 0.951)
ZHFA^*a*^	0.884 (−1.52 to 3.36)	−2.70 (−5.09 to 0.650)	−2.03 (−3.61 to –0.315)	−1.88 (−3.04 to 1.89)
Serum creatinine (mg/dL)	0.25 (0.2–0.46)	0.30 (0.20–0.40)	0.4 (0.2–0.6)	NA
Creatinine clearance^*b*^ (mL/min/1.73 m^2^)	124 (67.0–161)	110 (74.3–173)	145 (90–289)	NA
CD4 count (cells/mm^3^)	644 (465–724)	1,011 (271–1,708)	156 (24–652)	1,174 (982–1,695)
Absolute neutrophil count (cells/mm^3^)	2,235 (860–3,388)	436 (1,697–6,787)	1,480 (476–8,721)	3,070 (1,500–2,700)

^*a*^ The WHO and Centers for Disease Control and Prevention tables were used for the calculation of *z* scores.

^*b*^ Calculated by modified Schwartz formula (28): $0.413\;\cdot \left(\frac{height\;\left(cm\right)}{serum\;creatinine\;\left(mg/dL\right)}\right)$.

^*c*^ d4T, stavudine; 3TC, lamivudine; ABC, abacavir; NA, not available; ZHFA, height-for-age *z* score; ZWFA, weight-for-age *z* score. Participant demographics are at first pharmacokinetic sampling (week 2).

The validation data set included six children aged 2 (0.83–3.0) years, with a weight of 11 (9.0–12.0) kg, and ZWFA of −1.06 (−1.85 to 0.950). Three out of the six children were classified as WHO HIV stage 4, while the rest were at stage 3. Neutropenia was observed in four of the children (one: grade 1, one: grade 2, and two: grade 4) (15). A total of 36 samples of rifabutin and des-rifabutin were available, and none of them were BLQ.

### Population pharmacokinetic model

#### Structural model

The final joint model for rifabutin and des-rifabutin had two-compartment disposition models for both parent and metabolite with first-order elimination, and absorption delay was best described by lag time (Fig. S4). To account for differences in body weight, allometric scaling was incorporated a priori on all clearance and volume of distribution parameters, with normalization to the median weight of 10 kg.

The elimination of rifabutin and its metabolite des-rifabutin was best modeled with three distinct parameters: inhibitable CYP3A4 pathway (representing the elimination clearance of rifabutin by CYP3A4 that is inhibited by co-administration of LPV/r), clearance conversion (indicating the conversion of rifabutin to des-rifabutin by AADAC), and clearance metabolite (representing the elimination clearance of des-rifabutin). Although the two clearance pathways of rifabutin, by CYP3A4 and by AADAC, would normally not be separable, here we were able to do so due to the effect of LPV/r to inhibit the CYP3A4 pathway. Re-parametrizing the model by employing clearance conversion and a separate clearance for the parent that is considered as fully inhibited by LPV/r led to a significantly enhanced model fit compared to estimating the fraction metabolized (*F*_M_) with only a single clearance parameter (∆AIC = −36.6, df = 1). Consequently, we were able to estimate the volumes of distribution for the metabolite, which had initially been fixed to the volumes of distribution for the parent compound. This is all under the assumption that 100% of the rifabutin eliminated by the clearance conversion is transformed into des-rifabutin.

For a typical child weighing 10 kg not taking LPV/r, the estimated [95% confidence intervals (CIs)] rifabutin inhibitable CYP3A4 pathway was 13.6 (9.22–19.1) L/h; clearance conversion was 16.2 (12.4–21.1) L/h; and des-rifabutin clearance was 106 (80.8–145.0) L/h.

Considering the complexity of the model, variability parameters were included parsimoniously, where most relevant and essential. Variability between subjects was accounted for by incorporating a common between-subject variability (BSV) random effect on the two clearance pathways of rifabutin (both rifabutin inhibitable CYP3A4 clearance and clearance conversion), and a separate BSV was included for des-rifabutin clearance. Between-occasion variability was estimated for all absorption parameters.

Parameter estimates of the final model, together with their precision, are presented in Table 2.

**TABLE 2:** Parameter estimates for the rifabutin-des-rifabutin model

Parameter	Typical value (95% CI)^*a*^	RSE^*e*^ (%)	Parameter variability, % CV^*b*^ (95% CI)^*a*^	RSE^*e*^ (%)	Shrinkage (%)
Rifabutin					
Clearance (Inhibitable CYP3A4 pathway) (L/h)^*c*^	13.6 (8.77–18.8)	18.6	BSV: 20.7 (15.6–26.0)	26.7	14
Clearance conversion (AADAC pathway) (L/h)^*c*^	16.2 (12.8–20.7)	12.7			
Central volume of distribution, *V*_C,P_ (L)^*c*^	185 (135–251)	16.3			
Peripheral volume of distribution, *V*_P,P_ (L)^*c*^	232 (171–313)	15.5			
Intercompartmental clearance, *Q*_P_ (L/h)^*c*^	25.1 (17.3–33.1)	15.7			
Bioavailability, *F*	1 fixed	–	BOV: 71.9 (59.5–85.1)	15.4	3
Absorption rate constant, Ka (1/h)	1.27 (0.810–2.14)	29.0	BOV: 118 (84–175)	24.1	11
Absorption lag time, Lag (h)	0.544 (0.338–0.805)	21.9	BOV: 74.8 (44.4–105.0)	32.6	42
Des-rifabutin					
Clearance metabolite (L/h)^*c*^	106 (82.1–142.0)	14.2	BSV: 30.8 (23.2–39.2)	26.0	8
Central volume of distribution, *V*_C,M_ (L)^*c*^	43.0 (25.9–64.4)	22.7			
Peripheral volume of distribution, *V*_P,M_ (L)^*c*^	241 (169–322)	16.2			
Intercompartmental clearance, *Q*_M_ (L/h)^*c*^	44.4 (31.5–63.3)	18.7			
Covariates					
LPV/r effect on bioavailability (%)	+158 (+105 to +239)	13.1			
LPV/r effect on rifabutin clearance (inhibitable CYP3A4 pathway) (%)	−100 fixed	–			
LPV/r effect on des-rifabutin clearance (%)	−76.6 (−79.8 to −73.1)	7.76			
ZWFA effect (each point below −3) on bioavailability^*d*^ (%)	−26.0 (−34.1 to −18.1)	15.9			
Age effect (≤3 years old) on Ka (%)	−72.3 (−83.3 to −57.1)	24.4			
Residual unexplained variability					
Rifabutin: proportional error (%)	18.8 (16.3–21.7)	6.84			
Rifabutin: additive error (µg/L)	10.1 (5.24–15.9)	29.3			
Des-rifabutin: proportional error (%)	10.8 (8.41–13.6)	12.3			
Des-rifabutin: additive error (µg/L)	11.6 (9.75–14.1)	9.62			
Correlation coefficient for measurement error (%)	28.2 (14.6–42.1)	24.4			

^*a*^ Confidence intervals were computed with sampling importance re-sampling on the final model.

^*b*^ Between-subject variability (BSV) and between-occasion variability (BOV) were obtained using $\sqrt{e^{\left(OM^{2}\right)}-1^{\;}}$ and reported as approximate % CV.

^*c*^ Allometric scaling with total body weight. Values are reported for median weight of 10 kg.

^*d*^ ZWFA effect on bioavailability per unit decrease in children with a ZWFA of <−3.

^*e*^ RSE, relative standard error.

#### Covariate model

Co-treatment with LPV/r had a major impact on various pharmacokinetic parameters. Specifically, it increased rifabutin bioavailability by 2.58-fold (1.93–3.46) (∆OFV = −45.7, df = 1, *P* < 0.001); it fully inhibited one of the clearance pathways of rifabutin which is mediated by the CYP3A4 pathway (∆OFV = −40.6, df = 1, *P* < 0.001); and it reduced des-rifabutin clearance by 76.6% (74.4–78.3), (∆OFV = −78.6, df = 1, *P* < 0.001).

Additionally, we found that ZWFA had an impact on rifabutin bioavailability (∆OFV = −27.1, df = 2, *P* < 0.001). It was observed that as ZWFA decreased below −3, corresponding to the WHO classification of severely underweight, there was an estimated decrease of 26.0% (17.9%–33.7%) in bioavailability for each unit decrease in ZWFA (*P* < 0.05, df = 2). Finally, the younger cohort, aged ≤3 years, were estimated to have a 72.3% (48.5%–85.7%) slower rate of absorption (*P* < 0.05, df = 1). Maturation was tested on all clearance parameters and did not show statistical significance (∆OFV = −1.52, df = 2, *P* > 0.05). According to the parameter estimates, complete maturation was achieved at 8 months, which is the youngest age in the data set. As a result, it was not incorporated into the model.

#### Effect of LPV/r co-treatment on rifabutin and des-rifabutin exposures

When comparing model-derived individual exposures without and with the co-administration of LPV/r in the <3-year-old patients who received both treatments, the geometric mean ratio (GMR) of rifabutin AUC_0–24h_ and *C*_max_ with LPV/r co-treatment versus without were 1.25 (95% CI 0.544–2.88) and 1.07 (95% CI 0.464–2.46), respectively. For des-rifabutin, during LPV/r co-treatment, AUC_0–24_ increased by almost fivefold, with a GMR of 4.86 (95% CI 2.11–11.2), while *C*_max_ increased by over threefold, with a GMR of 3.52 (95% CI 1.53–8.10).

#### External validation

Validation of the base model with external data [from Moultrie et al. (15)], incorporating the effects of LPV/r, demonstrated the model’s effectiveness in accurately predicting these data (Fig. S5).

#### Model diagnostics

**Fig 1:**
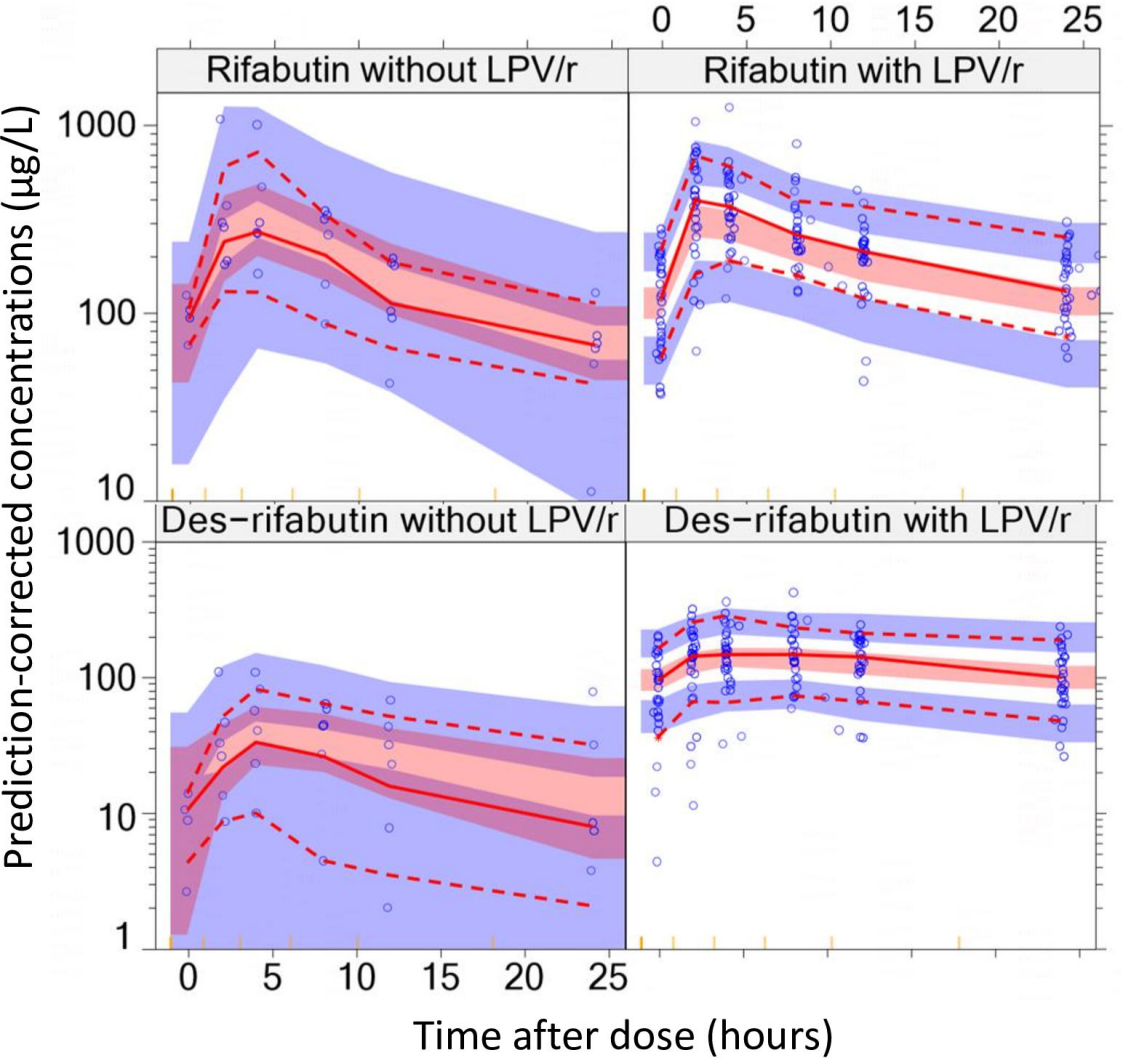
Prediction-corrected visual predictive check of the final rifabutin-des-rifabutin model. Blue circles represent observed plasma concentrations. The solid red line in the middle represents the median observed concentration, while the dashed red lines below and above it represent the 10th and 90th percentiles of the observed concentrations, respectively. The shaded area around each line represents the 95% model-predicted confidence intervals for the same percentiles. The yellow ticks at the base of the plot show the bins.

The pcVPC for rifabutin and des-rifabutin, stratified by presence or absence of LPV/r co-treatment, shows that the 10th, 50th, and 90th percentiles of the data are consistent with the respective 95% confidence intervals from the model, indicating good fit (Fig. 1).

### Simulation results

**Fig 2:**
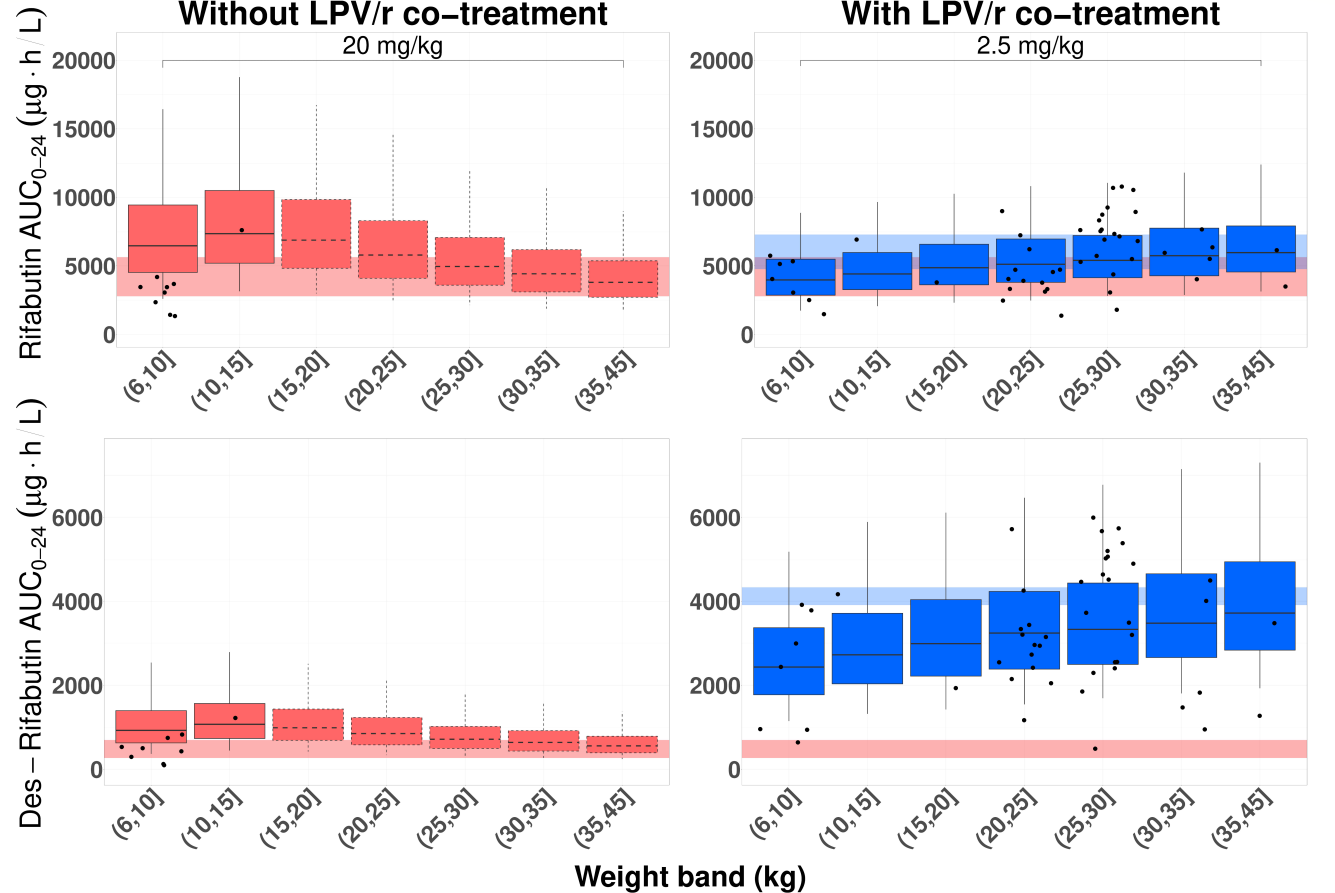
Simulated AUC_0–24h_ for rifabutin and des-rifabutin with the doses used in the study. Boxplots with dashed edges show weights which were not observed in the study, while the dots are model-derived AUCs for the study patients. Rifabutin dose without LPV/r co-treatment has been limited to 300 mg OD. Shaded areas indicate the median steady-state AUC_0–24h_ reported in adults (gray for 300 mg OD rifabutin without lopinavir/ritonavir, blue for 150 mg OD with lopinavir/ritonavir). The blue-shaded area for des-rifabutin is ±5% of reported AUC_0–24h_ values in adults for visualization purposes. The boxes indicate the interquartile range, while the whiskers show the 5th and 95th percentiles.

Simulations with the doses used in the study showed that the rifabutin dose used during TB-only treatment resulted in higher median pediatric exposures compared to median adult exposures for both rifabutin (9, 37) and des-rifabutin (9, 10) in the weight range of 6–20 kg (Fig. 2; Fig. S8). The highest exposure increase is in the 10- to 15-kg weight category, with a 30% increase for rifabutin and a 53% increase for des-rifabutin compared to the upper limit of adult median exposures (5,640 µg·h/L for rifabutin and 700 µg·h/L for des-rifabutin). During co-treatment with LPV/r, where the study dose was 2.5 mg/kg/day for the 1- to 3- and the 3- to 15-year-old cohorts, median pediatric exposures aligned with median adult exposures for both rifabutin and des-rifabutin (9, 10). For the cohort using a dose of 5 mg/kg/day in the study, weight groups of >7 kg exhibited higher median exposures, reaching up to 12% for rifabutin and 23% for des-rifabutin compared to the upper limit of median adult exposures during LPV/r co-treatment (7,290 µg·h/L for rifabutin and 4,130 µg·h/L for des-rifabutin) (see Fig. S8). Moreover, with the study doses, median *C*_max_ for both rifabutin and des-rifabutin remained below 900 µg/L (31–33) (see Fig. S6 and S9).

**Fig 3:**
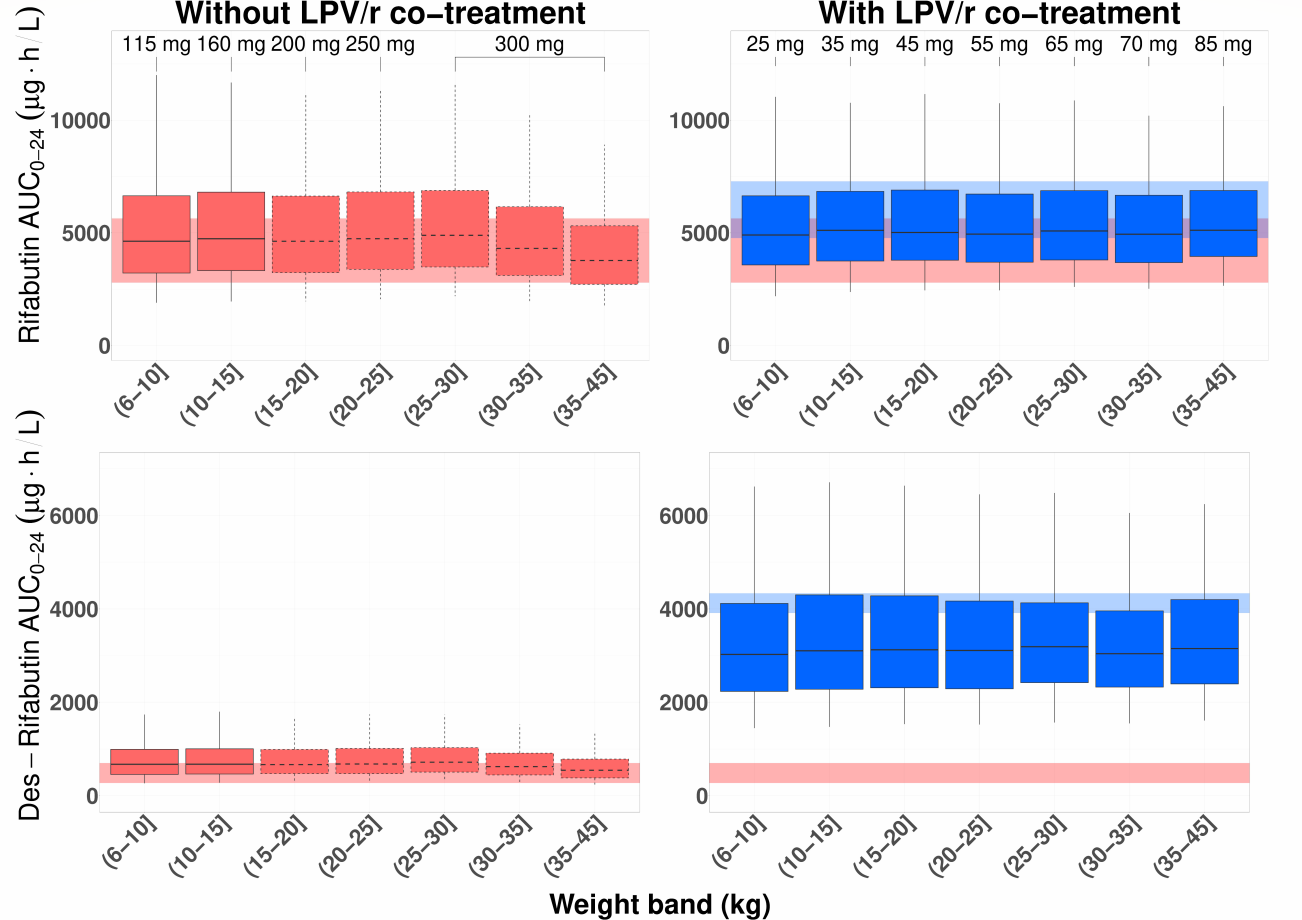
Simulated AUC_0–24h_ for rifabutin and des-rifabutin with suggested weight band-based doses (left panels: without LPV/r co-treatment, right panels: with LPV/r co-treatment). Shaded areas indicate the median steady-state AUC_0–24h_ range in adults (gray for 300 mg OD without lopinavir/ritonavir, blue for 150 mg OD with lopinavir/ritonavir). Boxplots with dashed edges show weights which were not observed in the study. The blue-shaded area for des-rifabutin is ±5% of reported AUC_0–24h_ values in adults for visualization purposes. The boxes indicate the interquartile range, while the whiskers show the 5th and 95th percentiles.

To improve upon the study doses and achieve consistent exposures across the weight bands, rifabutin doses were optimized using harmonized weight bands with and without LPV/r co-treatment, as shown in Table 3. With these doses, median rifabutin and des-rifabutin exposures are in line with those reported in adults both during standard TB treatment and during LPV/r co-treatment (Fig. 3). Additionally, median peak concentrations remain below 900 µg/L across all weight bands and treatment groups (Fig. S7).

**TABLE 3:** Optimized rifabutin doses for harmonized weight bands

	Rifabutin dose (mg)	
Harmonized weight bands (kg)	Without LPV/r co-treatment	With LPV/r co-treatment
≥6 to <10	115	25
≥10 to <15	160	35
≥15 to <20	200	45
≥20 to <25	250	55
≥25 to <30	300	65
≥30 to <35	300	70
≥35 to <45	300	85

## DISCUSSION

In this study, we used a model-based approach to characterize the population pharmacokinetics of rifabutin and des-rifabutin during co-treatment with LPV/r in children with TB and HIV. The co-administration of LPV/r significantly increases exposures of rifabutin and des-rifabutin by increasing the bioavailability and decreasing the clearance of both rifabutin and des-rifabutin. Furthermore, those severely underweight were associated with lower exposures due to a decreased bioavailability, and younger children were found to have slower absorption of rifabutin. In the context of LPV/r co-treatment, model-derived rifabutin and des-rifabutin exposures were higher compared to exposures without LPV/r co-treatment, in line with previously reported exposures in adults (9, 10). We performed Monte Carlo simulations to evaluate rifabutin and des-rifabutin exposures with the milligram per kilogram doses used in the studies. During LPV/r co-treatment, the rifabutin dose of 2.5 mg/kg/day resulted in simulated exposures in line with those of adults, while the dose of 5 mg/kg/day resulted in higher exposures than adults for children weighing >7 kg. During TB-only treatment, at a rifabutin dose of 20 mg/kg/day up to 300 mg/day, simulated exposures were higher than those reported in adults with 6- to 20-kg weights. We propose weight band-based dosing, using harmonized weight bands, showing that with these doses, pediatric exposures are consistent with those of adults across all weights and both treatment scenarios (without and with LPV/r co-treatment).

Hennig et al. developed a two-compartment model for rifabutin and des-rifabutin based mainly on data from adults, incorporating the impact of ritonavir-boosted protease inhibitor (PI) interactions (38). Our results align with their findings, indicating that co-administration of LPV/r significantly decreases rifabutin clearance through non-des-rifabutin routes by up to 100% and reduces des-rifabutin clearance by up to 76%. Our inhibitable rifabutin clearance extrapolated to a 70-kg adult of 58.5 L/h corresponds closely to their reported value of 58.8 L/h. However, our estimates of rifabutin clearance conversion and des-rifabutin clearance are higher than their reported values. On the other hand, they estimated higher steady-state volumes for both rifabutin and des-rifabutin, and while we found higher bioavailability with LPV/r co-treatment, they reported lower bioavailability during co-treatment with ritonavir-boosted PIs in TB/HIV patients. Their study consisted of data from adults, including a mix of various PIs, not limited to lopinavir, which could contribute to differences in the estimates.

High variability was observed in rifabutin bioavailability, which could be due to its complex pharmacokinetics. Rifabutin is partially metabolized by CYP3A4, present in both the gut and the liver (causing a significant first-pass effect), and partially by AADAC to des-rifabutin. Various factors such as enzyme maturation, age-related differences in CYP3A4 expression, and difference in absorption speed due to variable gastrointestinal motility between children could cause this (41). Another element contributing to the high variability in bioavailability may stem from variability in adherence or medication intake, a notable concern in the administration of pediatric drugs (42). In the conducted studies, rifabutin was administered as a suspension compounded from capsules, and inadequate shaking prior to drug administration, leading to settling of the medication or the child partially swallowing the medication, could cause lower drug concentrations. This could also potentially explain the low concentrations found after an unobserved dose compared to the concentrations after an observed dose. During modeling, we handled these low trough concentrations by the method of initialization described by Dansirikul et al. (24), as detailed in the supplemental material.

Monte Carlo simulations were conducted to assess the doses utilized in the study, comparing the simulated exposures with literature findings in adults. Simulations showed that the study dose used during standard TB treatment (20 mg/kg/day) results in median rifabutin and des-rifabutin exposures exceeding those of adults in children with 6- to 20-kg weights, while the dose of 2.5 mg/kg/day used during LPV/r co-treatment was adequate, resulting in median exposures of both rifabutin and des-rifabutin which matched adult exposures. However, the dose of 5 mg/kg/day during LPV/r co-treatment resulted in higher exposure than those of adults in children with >7-kg weights. Median rifabutin *C*_max_ remained below the toxicity threshold of 900 µg/L for both scenarios simulated, i.e., during TB treatment alone and with LPV/r co-treatment, including in doses that resulted in increased exposures. We performed further simulations proposing rifabutin pediatric dosing by harmonized weight bands to achieve median exposures that match those of adults consistently across the weights. Currently, there are no specific rifabutin formulations designed for pediatric use. The studies that informed this analysis utilized oral suspensions (20 mg/mL) compounded from Mycobutin^®^ capsules. The limited availability of pediatric data may account for the absence of dedicated formulations. Using the available data from these pediatric studies, our study could provide a potential step toward the development of rifabutin formulations tailored to the needs of pediatric patients.

Our study was limited by the absence of sufficient data for the cohort below 1 year old. The study in this cohort was stopped after recruitment of three patients, the youngest of whom was 8 months old, since the formulation was no longer available. The impact of maturation on rifabutin and des-rifabutin clearances was deemed insignificant in our data, with complete maturation estimated at 8 months in the model. Additionally, when exploring maturation on all clearance parameters using the maturation function reported for midazolam, a CYP3A4 probe, it was observed that rifabutin and des-rifabutin clearances did not follow the same pattern (27). Thus, simulation results from this study are applicable to children above 8 months old.

Based on WHO 2021 guidelines, LPV/r-based ART is considered an alternative first-line regimen for children (at least 4 weeks old and under 10 years old) and is a preferred second-line regimen for all children and adolescents who fail non-PI-based ART (4). Rifabutin is recommended in the context of HIV co-treatment, highlighting the importance of optimizing its dose in pediatric use. We have established a population pharmacokinetic model for children undergoing LPV/r co-treatment, offering a tool for rifabutin dose optimization. We suggest weight-band dosing, which aligns rifabutin and des-rifabutin exposures across age groups and exposures in adults. Further investigations are warranted to explore the relationship between rifabutin and des-rifabutin exposures and potential adverse effects.

## Data Availability

The data that support the findings of this study are available from the corresponding authors of the original studies upon reasonable request.

## SUPPLEMENTAL MATERIAL

Supplemental material aac.00354-24-s0001.docx Supplemental text on initialization; Figures S1 to S9.

ASM does not own the copyrights to Supplemental Material that may be linked to, or accessed through, an article. The authors have granted ASM a non-exclusive, world-wide license to publish the Supplemental Material files. Please contact the corresponding author directly for reuse.
