# Supplementary material for: Population pharmacokinetics of rifabutin among HIV/TB co-infected children on lopinavir/ritonavir-based antiretroviral therapy
Source: Antimicrob Agents Chemother. 2024 Jul 22;68(8):e00354-24. doi: 10.1128/aac.00354-24 (PMC11304744; doi:10.1128/aac.00354-24)
Supplement: Supplemental material — Supplemental text on initialization; Figures S1 to S9. [file aac.00354-24-s0001.docx]

**Population pharmacokinetics of rifabutin among HIV/TB co-infected children on lopinavir/ritonavir-based antiretroviral therapy**

Manna Semere Gebreyesus^1^, Roeland E.Wasmann^1^, Helen McIlleron^1^, Regina Oladokun^2^, Prosper Okonkwo^3^, Lubbe Wiesner^1^, Paolo Denti^ǂ1^, Holly E. Rawizza^ǂ4,5^

^1^Division of Clinical Pharmacology, Department of Medicine, University of Cape Town, Cape Town, South Africa

^2^Faculty of Clinical Sciences, Department of Pediatrics, College of Medicine, University of Ibadan, Nigeria

^3^APIN Public Health Initiatives (APIN), Abuja, Nigeria

^4^Brigham and Women’s Hospital, Boston, Massachusetts

^5^Harvard T.H. Chan School of Public Health, Boston, Massachusetts, USA

ǂAuthors with equal contribution

**Corresponding author:** Paolo Denti, Professor of Pharmacometrics

K45 Old Main Building, Groote Schuur Hospital Observatory, Cape Town, 7925, South Africa

Email: [paolo.denti@uct.ac.za](mailto:paolo.denti@uct.ac.za)

Phone: +27 21 404 7719

**Supplementary methods and results**

Table of Contents

[**Initialization** 3](#_Toc159518007)

[**List of supplementary figures** 4](#_Toc159518008)

[Figure S1. 4](#_Toc159518009)

[Figure S2. 5](#_Toc159518010)

[Figure S3. 6](#_Toc159518011)

[Figure S4. 7](#_Toc159518012)

[Figure S5. 8](#_Toc159518013)

[Figure S6. 9](#_Toc159518014)

[Figure S7. 10](#_Toc159518015)

[Figure S8. 11](#_Toc159518016)

[Figure S9. 12](#_Toc159518017)

# **Initialization**

There were fifteen profiles in our data across different age cohorts and visits where the trough concentration (sample collected at pre-dose from unobserved dose) was unexpectedly low compared to the 24-hour concentration (Figure S3 illustrates an example of such a profile). Rifabutin is expected to reach steady state within 10 days and in this study had been dosed for two weeks prior to sampling at each visit, therefore it would be expected that trough concentrations would be in line with the 24-hour concentrations. Model fit was inadequate for these trough concentrations, as observed by the visual predictive check, since model predictions were higher than the observations for those time points. These profiles were handled by initialization with the B2 method as suggested by Dansirikul *et al*. (1), where the doses are discarded and the observed trough concentrations are used as a starting point (or the model is initialized to the first observation) with residual error to account for measurement error. Parameter estimates improved after initialization in terms of stability, and the additive error decreased (from 15.0 to 11.3 µg/L for rifabutin and from 13.5 to 10.7 µg/L for des-rifabutin after initialization). Moreover, the high variability which had been estimated for bioavailability and rifabutin inhibitable CYP3A4 clearance was reduced (between-occasion-variability (BOV) of bioavailability decreased from 103% to 66.7% after initialization and between-subject-variability (BSV) of rifabutin inhibitable CYP3A4 clearance decreased from 204% to 36.3%). Initialization improved the model fit, as observed by the pcVPC (Figure 1). This confirms that the trough concentrations did not fit with the dosing information, possibly due to a lack of adherence.

# **List of supplementary figures**


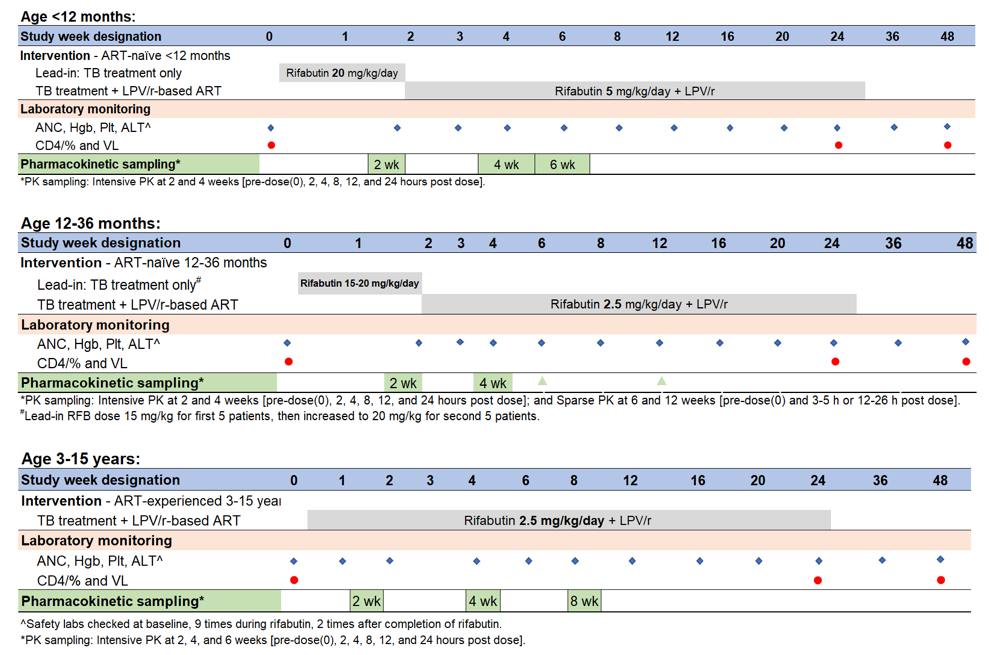


Figure S1. Study design for the three cohorts from which data were obtained for this analysis.


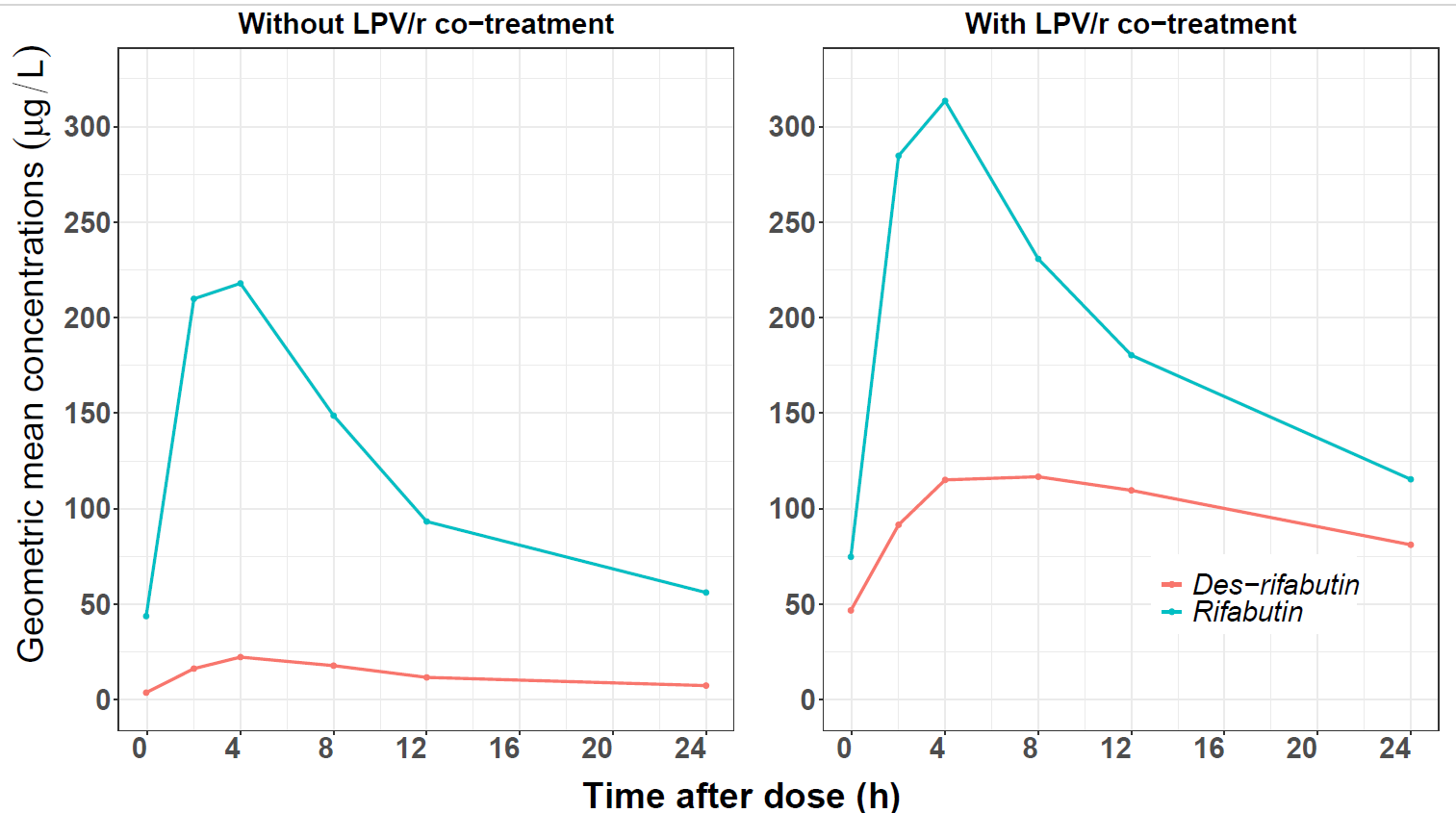


Figure S2. Summary (geometric mean) concentration-time profiles of rifabutin and des-rifabutin without and with lopinavir/ritonavir (LPV/r) co-treatment in the original data used for modelling. Without LPV/r co-treatment = data from <1-year-old and 1-to-3-year-old cohorts; With LPV/r co-treatment = data from <1-year-old, 1-to-3-year-old, and 3-to-15-year-old cohorts.


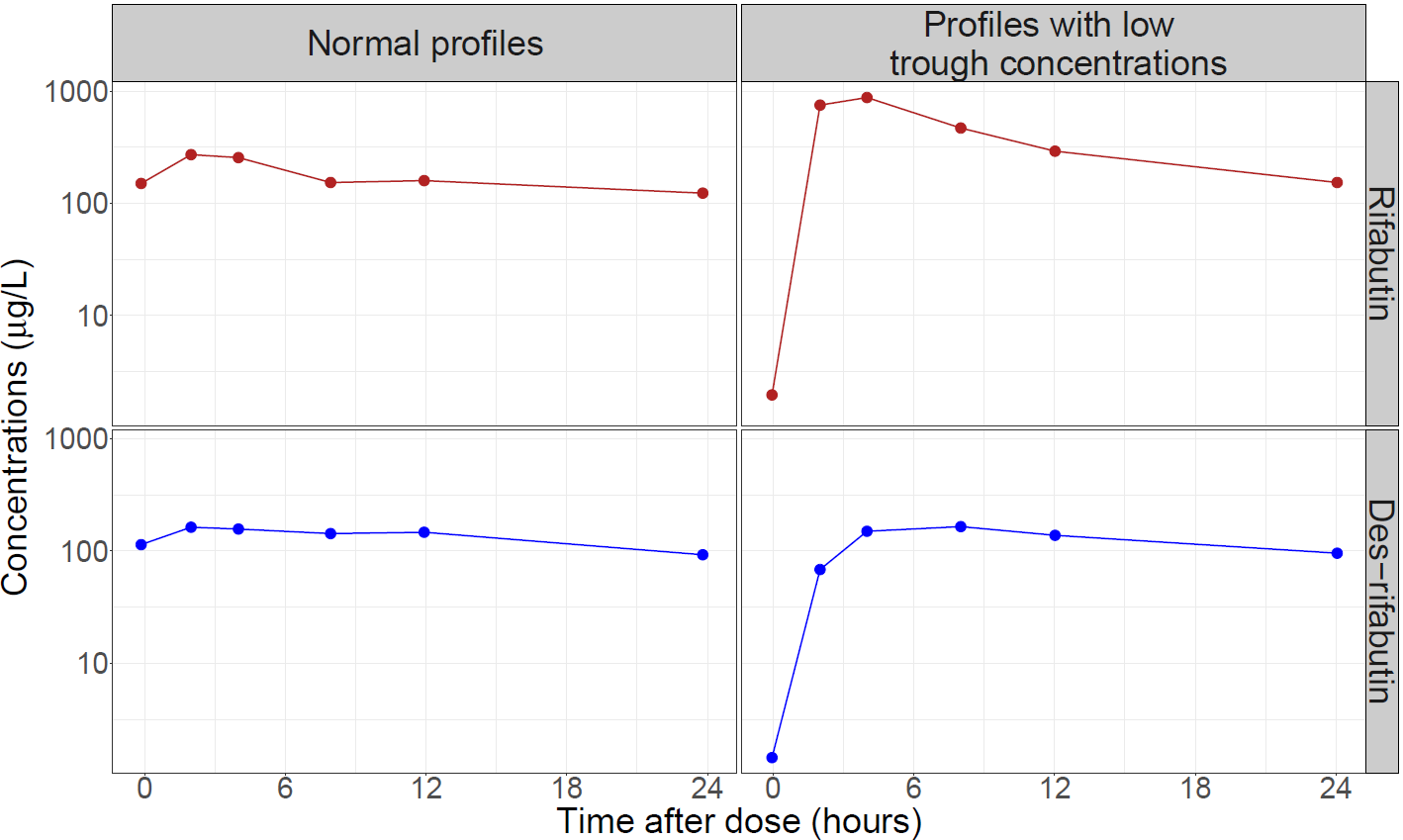


Figure S3. Raw data normal profiles (left panels) and profiles with low trough concentrations (right panels).


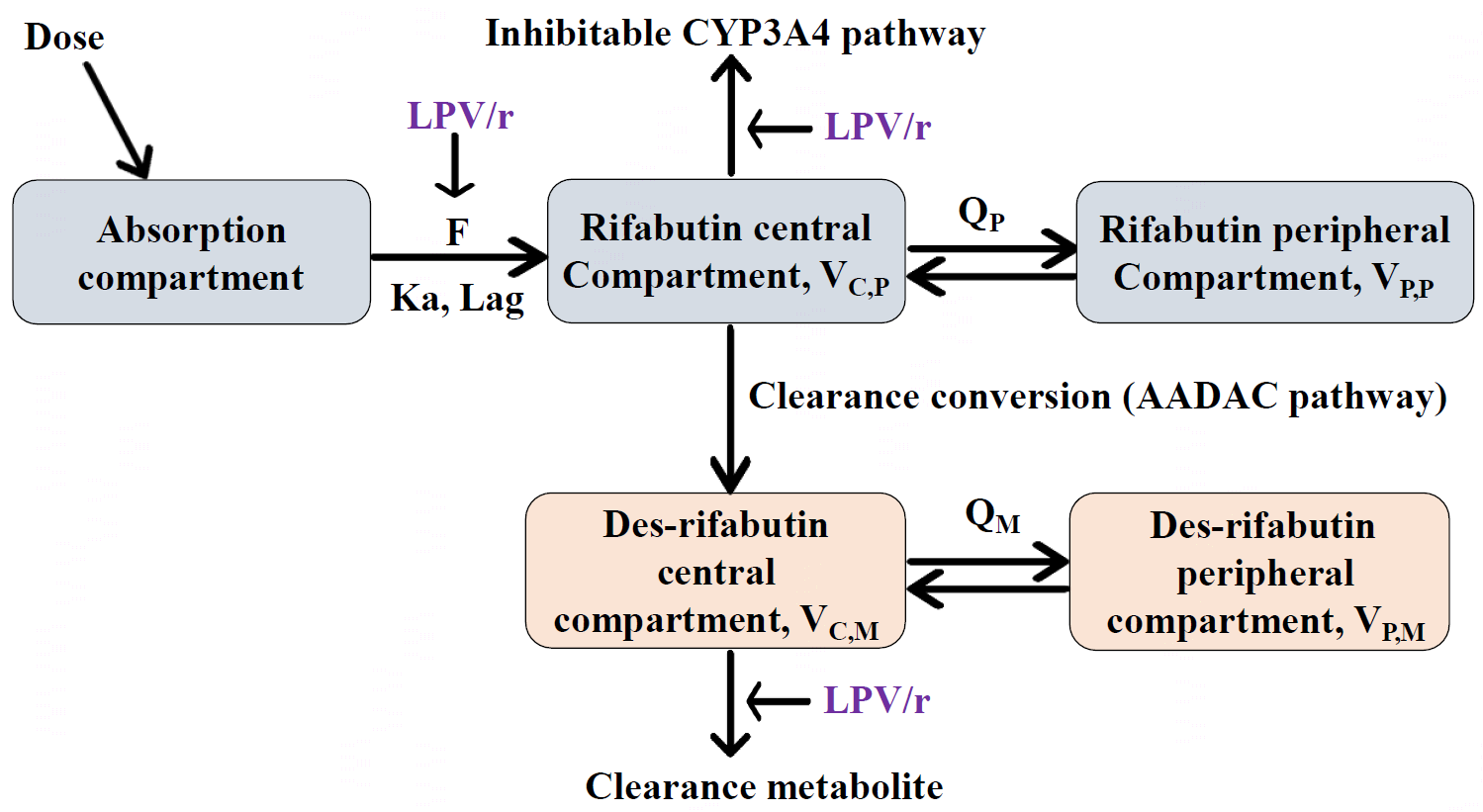


Figure S4. Model schematic for the final joint population pharmacokinetic model of rifabutin and des-rifabutin with lopinavir/ritonavir co-treatment. Bio, oral bioavailability; Ka, first-order absorption rate constant; Lag, absorption lag time; Q_P_, parent intercompartmental clearance; Q_M_, metabolite intercompartmental clearance; LPV/r, lopinavir/ritonavir; AADAC, arylacetamide deacetylase.


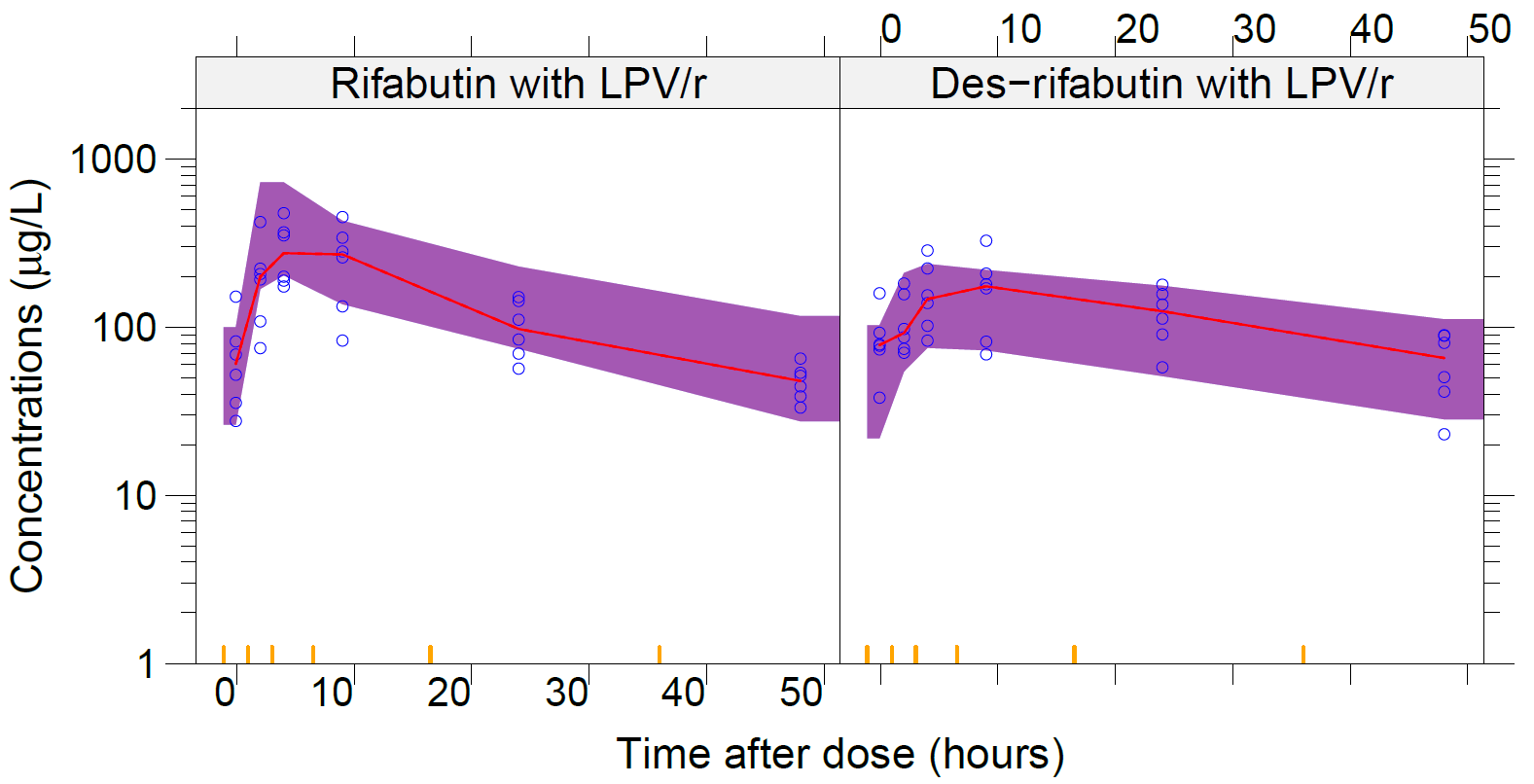


Figure S5. External model validation using data from six South African children by Moultrie et al. The solid line in the middle represents the median observed concentration while the shaded area around the line represents the 95% model-predicted confidence interval for the median. The yellow ticks at the base of the plot show the bins.


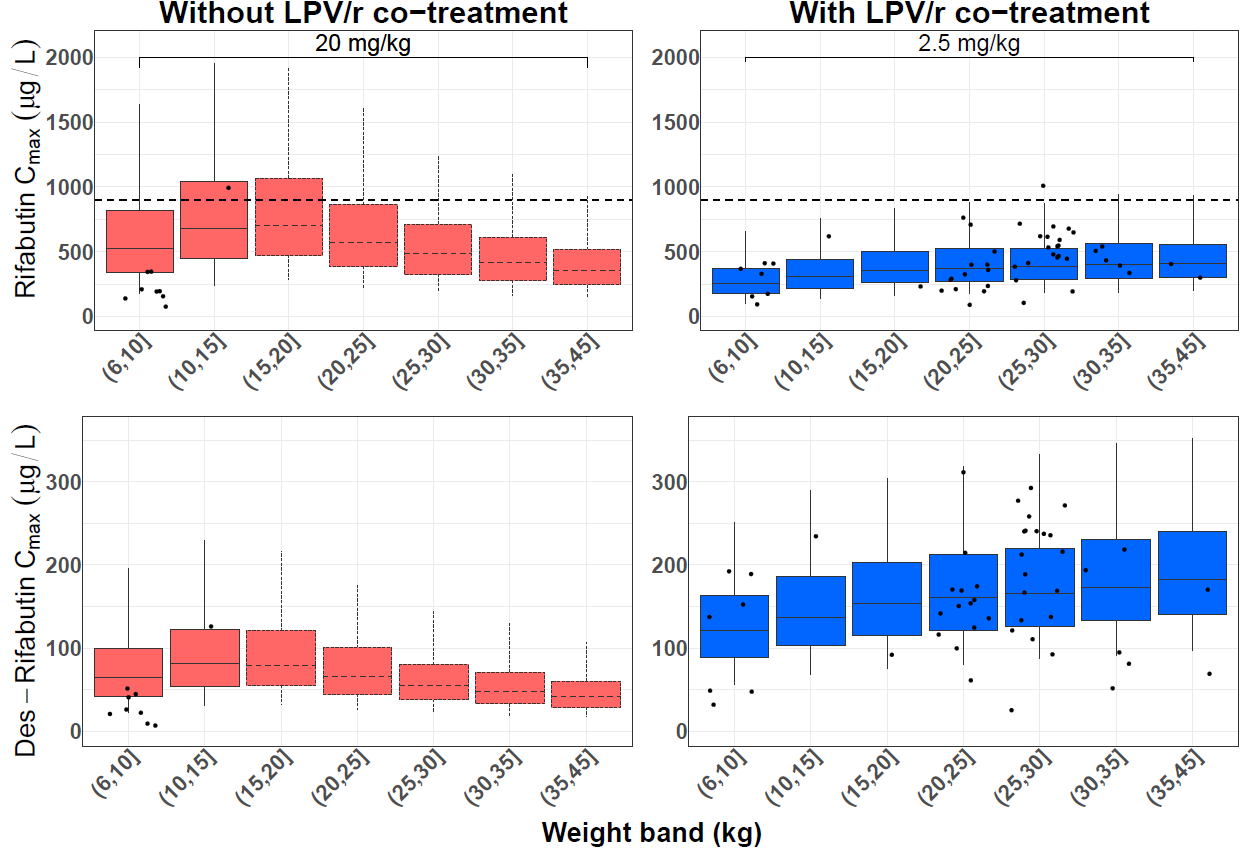


Figure S6. Simulated C_max_ values for rifabutin and des-rifabutin with the study doses. The black dashed line shows C_max_ value of 900 µg/L, which is a toxicity limit for rifabutin. Boxplots with dashed edges show weights which were not observed in the study while the dots are model-derived C_max_ values for the study patients. The boxes indicate the interquartile range, while the whiskers show the 5^th^ and 95^th^ percentiles.


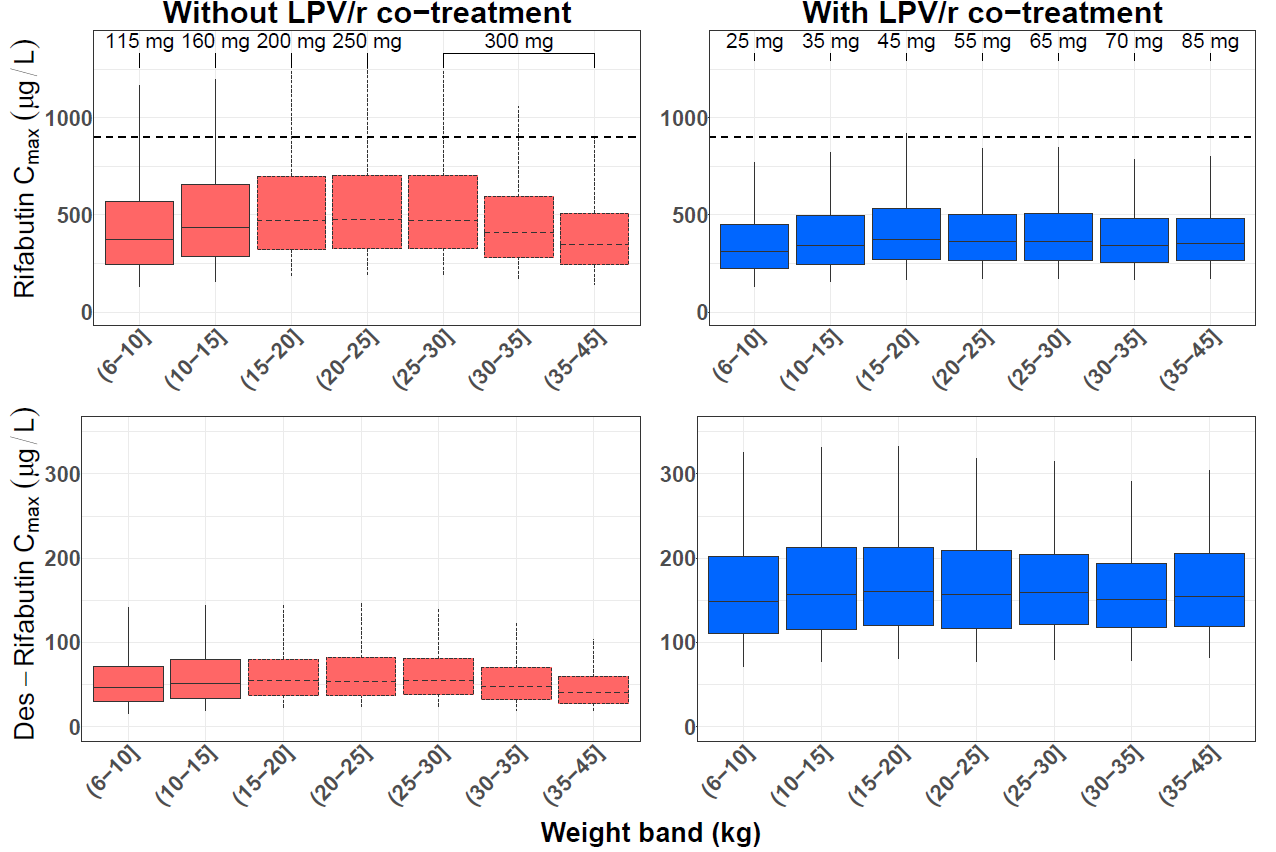


Figure S7. Simulated C_max_ values for rifabutin and des-rifabutin with suggested weight-band based doses. The black dashed line shows C_max_ value of 900 µg/L, which is a toxicity limit for rifabutin. Boxplots with dashed edges show weights which were not observed in the study. The boxes indicate the interquartile range, while the whiskers show the 5^th^ and 95^th^ percentiles.


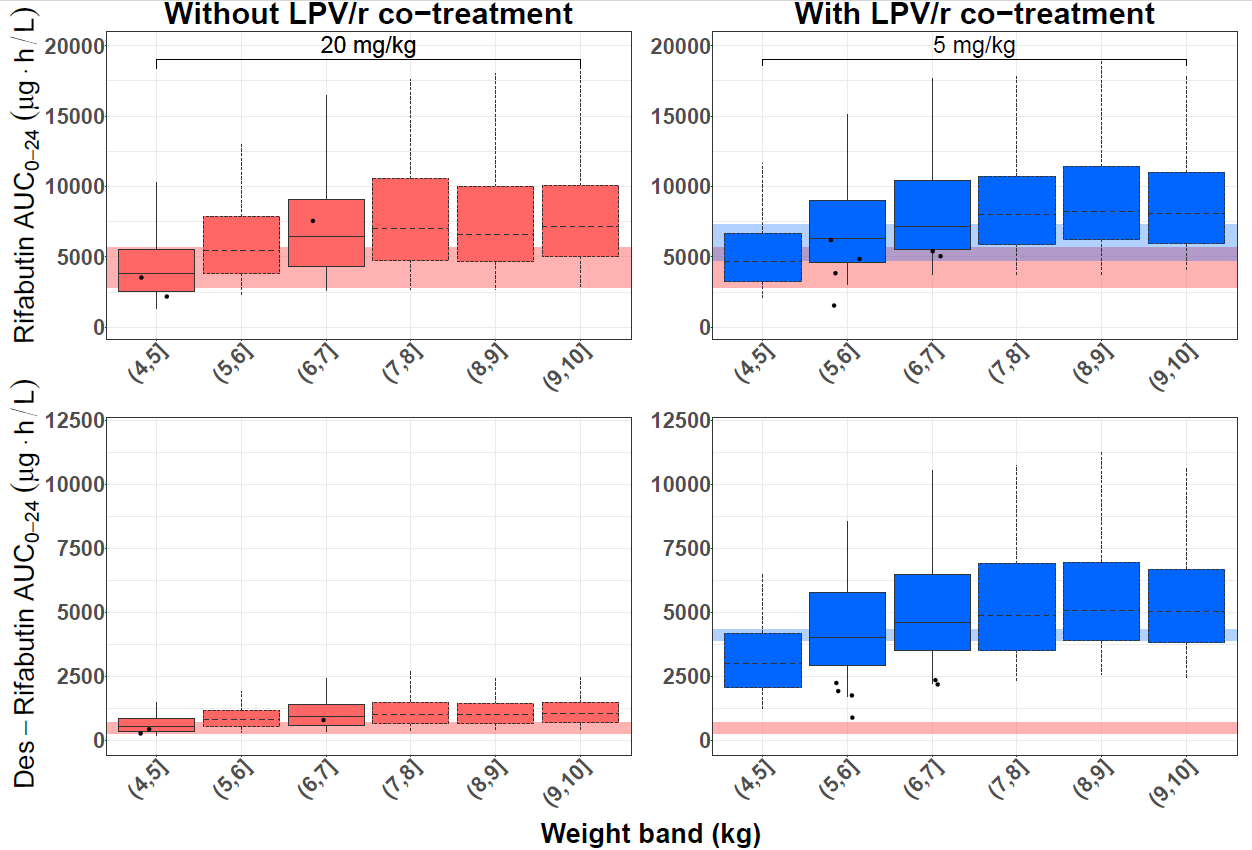


Figure S8. Simulated AUC_0-24_ values for rifabutin and des-rifabutin with the doses used in the study for the <1-year-old cohort**.** Shaded areas indicate the median steady-state AUC_0-24_ range in adults (grey for 300 mg OD without lopinavir/ritonavir, blue for 150 mg OD with lopinavir/ritonavir). Boxplots with dashed edges show weights which were not observed in the study while the dots are model-derived AUCs for the study patients. The boxes indicate the interquartile range, while the whiskers show the 5^th^ and 95^th^ percentiles.


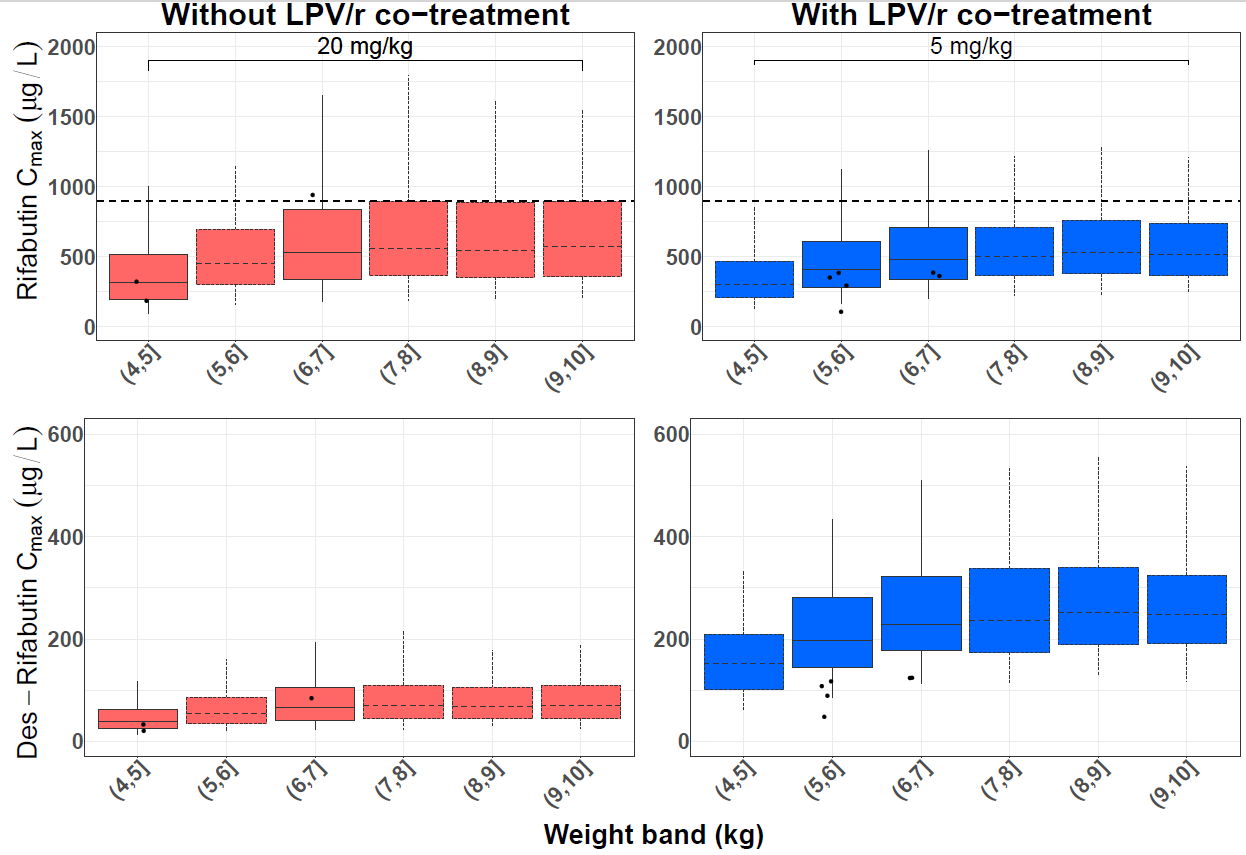


Figure S9. Simulated C_max_ values for rifabutin and des-rifabutin with the doses used in the study for the <1-year-old cohort**.** The black dashed line shows C_max_ value of 900 µg/L, which is a toxicity limit for rifabutin. Boxplots with dashed edges show weights which were not observed in the study while the dots are model-derived AUCs for the study patients. The boxes indicate the interquartile range, while the whiskers show the 5^th^ and 95^th^ percentiles.
